# Bilateral Facial Paralysis

**Published:** 1908-02-29

**Authors:** 


### BILATERAL FACIAL PARALYSIS.

The following case exemplifies two things, namely bilateral paralysis of both seventh and both eighth cranial nerves, and the wonderful degree to which some patients, when they have the will, can make the very best of their troubles.

The case further offers scope for speculation as to the cause of the lesion. It may have been bilateral otitis media with infection of both aqueducts of Fallopius, though it is well known that lesion of the facial nerve is remarkably uncommon in cases of otitis media, apart from accident, so that to have it on both sides in the same patient must be extremely rare. Another possibility is that he had bilateral cholesteatomata of the brain close to the internal auditory meati. If any reader has any other suggestions to offer as to the nature of the trouble, the editors will be glad to discuss them.

The man, a water-cress gatherer, came under observation for a gouty effusion into his left knee joint when he was 42 years old. He was perfectly deaf, and his face was paralysed on both sides to a remarkable extent.

It appeared that the illness which issued in this formidable paralysis of the face began 13 years ago. He was then seized with a severe pain in the left foot, so bad that he could not put his foot to the ground that day. In the night he was attacked with violent pain in the forehead, and that in the foot left him. Soon after this he appears to have become paralysed on the right side of the face—the eye, as he describes it, staring open, and looking large and prominent. He used to suffer from pains in the head, chiefly on the right side, and in the right ear, with more or less of noise in the head. His hearing now became affected, at first so that talking to him was exquisitely painful, but afterwards deafness came on, affecting both ears.

A discharge now came from the right ear, and he describes the lower lip as "dropping down," and the jawbone on both sides appearing out of place. Probably both buccinator muscles became paralysed at this time. When he ate or spoke he was forced to put his hand to his lower lip to hold it up.

The following is a description of his condition at the present time:—

The features generally lack expression, and their muscles are wasted. The eyes appear to protrude, and the eyelids are farther apart than natural, so that a large portion of the sclerotic is thus exposed to view. The conjunctivæ are suffused with tears, and slightly injected. Winking takes place at about the usual intervals, though less completely than natural. The patient can approximate the lids voluntarily when told to shut his eyes, but he cannot bring them into contact one with the other, and in endeavouring to do this the eyes are rolled strongly upwards until the corneæ disappear under the upper lids. The man can neither frown nor raise the eyebrows, nor laugh, nor whistle, nor put in action any of the muscles supplied by the seventh cranial nerves, unless it be the orbicularis palpebrarum to the extent already indicated. It is more than probable that the partial closure of the lids is due rather to a voluntary relaxation of the levator palpebræ superioris than to a contraction of the orbicularis palpebrarum. The upper lip is so elongated that its border is on a level with the margin of the gum of the lower jaw, while the lower lip is everted and pendant as if a weight were suspended from its free edge. By an inspiratory effort both lips can be brought into apposition, and maintained so as long as the forcible inspiration is continued.

The cheeks are wasted, sunken, and flaccid, and the patient has lost the greater number of his teeth. The paralytic condition of the lips and cheeks, together with the loss of teeth, renders the pronunciation of labials and dentals impossible; consequently the man's articulation is very imperfect, and mastication is performed with some difficulty. He is obliged to eat slowly, and to make use of his fingers in eating and in removing accumulations of food.

In drinking, he presses the rim of the glass against the cuticular surface of the lower lip just below its mucous margin, whereby the pendulous free edge is lifted up and bent inwards towards the cavity of the mouth. In consequence of the lips being always apart, the labial glands on the lower lip can be seen to pour out their clear fluid secretion, which stands in minute and separate drops, like fine dew covering its surface.

The patient is perfectly deaf in both ears, the loudest sounds, even through an ear-trumpet, being quite inaudible to him. Notwithstanding this high degree of deafness, he fancies he can hear, and he is a regular attendant at church. He says he can hear the playing of the organ and the rumbling of carriages in the street. He certainly is conscious of the passing of vehicles; but it is evident that he derives the information from the vibrations conveyed to the general surface of his body and not to the auditory organs; in other words he feels the vibrations, but does not hear them. It is probable, therefore, that he is only conscious of the lower notes upon the organ, and would not detect that the organ was being played if all the notes struck were in the treble.

He has never had paralysis of any other cranial nerve; no diplopia, nor other ocular lesion to suggest syphilis; nor are the hypoglossal nerves affected, so that an extension from bulbar paralysis does not seem likely. The trouble is stationary, and long has been. The fifth nerves are normal both as to their sensory and their motor functions. Taste ought to be impaired, but it is not lost, and the patient does not complain of any loss of power to taste things at all. There is no nystagmus, nor any ataxia suggestive of a cerebellar lesion.

He has occasional tinnitus which he likens to the noise of putting a large sea-shell to the ear, and sometimes he hears a kind of hissing noise; sudden snappings are likewise occasionally heard in the left ear. On examining the ears with the otoscope, nothing abnormal is detected on the left side; but from the right meatus there flows, in small quantity, a fœtid, purulent, and occasionally bloody, discharge. Granulations growing from the cavity of the tympanum occupy the bottom of this passage, the membrana tympani being destroyed and the ossicles gone.

